# *Prokaryotic-*virus-encoded auxiliary metabolic genes throughout the global oceans

**DOI:** 10.1186/s40168-024-01876-z

**Published:** 2024-08-29

**Authors:** Funing Tian, James M. Wainaina, Cristina Howard-Varona, Guillermo Domínguez-Huerta, Benjamin Bolduc, Maria Consuelo Gazitúa, Garrett Smith, Marissa R. Gittrich, Olivier Zablocki, Dylan R. Cronin, Damien Eveillard, Steven J. Hallam, Matthew B. Sullivan

**Affiliations:** 1 Department of Microbiology, Ohio State University, Columbus, OH 43210 USA; 2 Center of Microbiome Science, Ohio State University, Columbus, OH 43210 USA; 3 EMERGE Biology Integration Institute, Ohio State University, Columbus, OH 43210 USA; 4 Centro Oceanográfico de Málaga (IEO-CSIC), Puerto Pesquero S/N, 29640 Fuengirola (Málaga), Spain; 5 Viromica Consulting, 8320000 Santiago, Chile; 6 Université de Nantes, CNRS, LS2N Nantes, France; 7 Research Federation for the Study of Global Ocean Systems Ecology and Evolution, R2022/Tara GO-SEE, Paris, France; 8 Department of Microbiology & Immunology, University of British Columbia, Vancouver, BC V6T 1Z1 Canada; 9 Graduate Program in Bioinformatics, University of British Columbia, Vancouver, BC V6T 1Z4 Canada; 10 Genome Science and Technology Program, University of British Columbia, 2329 West Mall, Vancouver, BC V6T 1Z4 Canada; 11 Life Sciences Institute, University of British Columbia, Vancouver, BC V6T 1Z3 Canada; 12 ECOSCOPE Training Program, University of British Columbia, Vancouver, BC V6T 1Z3 Canada; 13 Department of Civil, Environmental, and Geodetic Engineering, Ohio State University, Columbus, OH 43210 USA; 14 Department of Medicine, The University of Chicago, Chicago, IL USA; 15 Biology Department, Woods Hole Oceanographic Institution, Woods Hole, MA USA

**Keywords:** Prokaryotic, AMGs, *Tara* Oceans

## Abstract

**Background:**

Prokaryotic microbes have impacted marine biogeochemical cycles for billions of years. Viruses also impact these cycles, through lysis, horizontal gene transfer, and encoding and expressing genes that contribute to metabolic reprogramming of prokaryotic cells. While this impact is difficult to quantify in nature, we hypothesized that it can be examined by surveying virus-encoded auxiliary metabolic genes (AMGs) and assessing their ecological context.

**Results:**

We systematically developed a global ocean AMG catalog by integrating previously described and newly identified AMGs and then placed this catalog into ecological and metabolic contexts relevant to ocean biogeochemistry. From 7.6 terabases of *Tara* Oceans paired prokaryote- and virus-enriched metagenomic sequence data, we increased known ocean virus populations to 579,904 (up 16%). From these virus populations, we then conservatively identified 86,913 AMGs that grouped into 22,779 sequence-based gene clusters, 7248 (~ 32%) of which were not previously reported. Using our catalog and modeled data from mock communities, we estimate that ~ 19% of ocean virus populations carry at least one AMG. To understand AMGs in their metabolic context, we identified 340 metabolic pathways encoded by ocean microbes and showed that AMGs map to 128 of them. Furthermore, we identified metabolic “hot spots” targeted by virus AMGs, including nine pathways where most steps (≥ 0.75) were AMG-targeted (involved in carbohydrate, amino acid, fatty acid, and nucleotide metabolism), as well as other pathways where virus-encoded AMGs outnumbered cellular homologs (involved in lipid A phosphates, phosphatidylethanolamine, creatine biosynthesis, phosphoribosylamine-glycine ligase, and carbamoyl-phosphate synthase pathways).

**Conclusions:**

Together, this systematically curated, global ocean AMG catalog and analyses provide a valuable resource and foundational observations to understand the role of viruses in modulating global ocean metabolisms and their biogeochemical implications.

Video Abstract

**Supplementary Information:**

The online version contains supplementary material available at 10.1186/s40168-024-01876-z.

## Introduction

Prokaryotic microbes (bacteria and archaea) in the global oceans comprise an interconnected metabolic engine driving coupled biogeochemical cycles that help to sustain conditions for life on Earth [1–3]. These microbes are influenced by viruses that modulate microbial community structure and metabolic function. On an average day, viruses lyse ~ 20–40% of bacterial cells in the sunlit surface oceans [4]. Beyond lysis, virus transduction events (estimated in Tampa Bay and extrapolated to the global ocean) move ~ 10^29^ genes per day from one host cell to another [5], which likely contributes to adaptation and response patterns within microbial food webs. Indeed, microbial cells undergo such drastic reprogramming during infection that virus-infected cells (termed “virocells”) are increasingly recognized as distinct entities that have altered internal metabolisms and external outputs [6–8].

Reprogramming occurs either through changes in the expression of regulatory proteins or targeting specific metabolic pathways with auxiliary metabolic genes (AMGs) encoded in virus genomes [3]. AMGs are virus-encoded genes that are acquired from hosts and sporadically present across phage genomes to a relatively unknown degree [9, 10]. Viruses are thought to randomly “sample” host DNA during infection and that a subset of these horizontal gene transfer events become “fixed” in the virus genome if they confer a fitness advantage [11, 12]. Virus lysis and metabolic reprogramming of prokaryotic cells alter nutrient cycling and carbon export, although the relevance of this for biogeochemical cycling is challenging to quantify, particularly at global ocean scales [11].

We reasoned that systematically cataloging and ecologically contextualizing AMGs could provide some inferences of the wider relevance of virus infection of prokaryotes in the global oceans. Ocean biogeochemical models have largely overlooked the role of viruses owing to a scarcity of empirical data. Hundreds of thousands of virus populations (approximate species-ranked taxa) are now cataloged in the global oceans [13–17], yet only four have metabolic reprogramming data available [10, 18–20]. By elucidating the frequency of viral populations carrying AMGs and the metabolic pathways they modulate, we hoped to fill this critical knowledge gap.

Culture-based model systems have revealed a lot about AMGs and their biology (Table S1). For example, marine cyanobacterial viruses (i.e., cyanophages) have been found to carry AMGs involved in photosynthesis, central carbon metabolism, phosphate acquisition, and nucleotide synthesis (Table S1), with a subset of these confirmed experimentally and shown to increase fitness [18]. Among the AMGs involved in photosynthesis are those encoding the D1 and D2 proteins of the photosystem II reaction center complex (*psbA* and *psbD*, respectively), as well as high-light-inducible proteins, plastocyanin, and ferredoxin [21–23]. The virus versions of these genes appear to evolve under different evolutionary selection pressures [24], which might create novel photosynthesis performance variants (e.g., a more stable D1 protein) for virus-infected cells. Such AMG-driven adaptations can impact cellular outputs, as seen with the reprogramming of photosynthesis in cyanobacteria, which increases carbon and energy fluxes into metabolic pathways co-opted for virus particle synthesis [18]. Phages in heterotrophic model systems also employ AMGs [25], with known examples including the *mazG* and *phoH* genes (Table S1 and S2). Whether the relative dearth of AMGs among the genomes of heterotroph-infecting phages is a function of lack of study (e.g., few available heterotrophic phages) or a real biological phenomenon (e.g., that cyanophages are enriched for AMGs) remains unknown.

Complementing culture-based inferences and experiments, metagenomic surveys have furthered our understanding of AMGs in the oceans (Table S2). These surveys have identified new AMGs associated with photosynthesis, carbon metabolism, nucleotide metabolism, nitrogen cycling, sulfur cycling, phosphorus cycling, vitamin B12 biosynthesis, and signaling pathways (Table S2). However, in silico AMG exploration remains challenging. For example, global cross-study comparisons are problematic given that identification and functional annotation are highly variable between studies; some use a single database (e.g., PFAM as in our work [14]), while others employ multiple databases (e.g., KEGG, PFAM, GhostKOALA, eggNOG, pVOG as in [26]). Moreover, quality control issues are ubiquitous, leading to cellular metabolic genes being mis-assigned to virus genomes (see re-analyses of early virome inferences in [27]). Even when long contigs are available (e.g., a provirus > 50 kb), metabolic genes in cellular genome regions can be misassigned as AMGs (see re-analysis of multidrug efflux pumps/resistance genes in [28]). Lastly, even where AMGs have been correctly identified and annotated, only 4 out of the 109 studies (in Table S1 and S2) have tried to integrate them into the context of cellular metabolic pathways [10, 18–20].

Fortunately, standardization, identification, and curation of AMGs have all gained traction in the literature [26, 29–33], and large-scale, highly curated, global-ocean datasets [14, 15, 34, 35] are now available to apply systematic approaches. Here, we leverage these improvements to explore 7.6 terabases of global *Tara* Oceans (TO) and *Tara* Oceans Polar Circle (TOPC) sequencing data derived from paired virus [14, 15] and prokaryote [35, 36] enriched metagenomic data sets. We modified an approach outlined previously [29], optimizing it for scalable data processing and analyses, to deliver each a permissive and conservative AMG catalog for the global oceans, which we then use to chart the metabolic, ecological, and biogeochemical context of virus AMGs in the global oceans.

## Materials and methods

### *Tara* Oceans expedition metagenome selection, curation, and virus identification

We established a global ocean dataset of 127 paired prokaryote-enriched and virus-enriched metagenomic datasets that were previously published [15, 35] (Table S3). These represented data from 62 stations during *Tara* Oceans (TO) and *Tara* Oceans Polar Circle (TOPC) expeditions, spanning across the South Pacific Ocean, North Pacific Ocean, South Atlantic Ocean, Indian Ocean, Red Sea, Mediterranean Sea, Southern Ocean, North Atlantic Ocean, and Arctic Ocean. Assembled contigs of the microbiome and virome datasets were processed with VirSorter2 [37] with a minimum score of 0.75 for the initial screening. Subsequently, contigs that were ≥ 5 kb and had the highest score from the “dsDNA phage” classifier when compared with other classifiers used in VirSorter2 [37] were considered possible viruses. After two passes through VirSorter2, with the prep-for-dramv flag to prepare for AMG identification, the remaining “possible virus” contigs were clustered into virus populations if they shared 95% average nucleotide identity across 80% of the genome as suggested previously [14, 38, 39] using MMSEQS2 (easy-cluster) [40].

### AMG identification and novelty

To systematically identify AMGs, we used our previously established protocol [29] but with additional curation steps. Briefly, all virus contigs were annotated using DRAM-v (v 1.0.6) [31] with a bit score ≥ 60. A gene was regarded as an AMG candidate if assigned to a metabolic module, and/or to a previously described AMG, and had an auxiliary score (confidence of virus-encoded) ≤ 3. This led to the establishment of a *permissive AMG catalog* (Fig. S1). To establish a *conservative AMG catalog*, we applied *post-DRAM-v curation rules* as follows. Firstly, to remove any non-virus region(s) on a contig, an AMG was only kept if it was located within virus regions called by CheckV (v 0.3.0) [28] and/or was found on a contig with a score ≥ 0.95 assigned by VirSorter2 [37]. Secondly, we screened for non-virus regions by checking for sequences adjacent to phage genome ends, including tRNA regions and inverted/direct repeats. tRNA regions were detected by tRNAscan-SE (v 1.23) [41] using general tRNA models. Inverted and direct repeats were predicted using the application inverted in EMBOSS (v 6.6.0) [42] with standard qualifiers and -scircular1 for circular virus contigs. For AMGs with predicted phage ends, only those that met the first two scenarios were kept (see 1 & 2 in the right panel of Fig. S1). Thirdly, we removed AMGs on virus contigs containing mobile genetic elements and other genes that may facilitate the random integration of microbial metabolic genes. AMGs were excluded if they were found on contigs carrying genes encoding transposons, lipopolysaccharide islands (glycosyltransferase, nucleotidyl transferase, carbohydrate kinases, and nucleotide sugar epimerase), endonucleases, integrases, or plasmid stability genes. Finally, we clustered the nucleotide sequences of AMGs in the conservative catalog into gene clusters using MMSEQS2 [40] with the following parameters: -cluster-mode 2 -cov-mode 1 -c 0.9 -s 7 -kmer-per-seq 20 as described in [30].

To establish the number of novel AMG sequences, we obtained previously reported AMG sequences or virus contigs from all published papers available as of November 2023. Of 101 publications reporting AMGs, 64 publications had virus contigs or AMGs sequences reported, or authors shared their AMG sequences. Where virus contigs were provided but AMG sequences were not (*n* = 58), we used our permissive AMG identification approach (described above) to identify AMGs. To formally, and systematically, estimate AMG novelty, we used a protein clustering approach. Specifically, we clustered all published AMG amino acid sequences (herein referred to as reference AMG sequences) with the 22,779 AMG sequences identified in our dataset using MMSEQS2 [40] with the following parameters -min-seq-id 0.3 -c 0.6 -s 7.5 as described in [30]. AMGs from our dataset that did not cluster with the published, reference AMG sequences were considered “novel.”

### Taxonomic annotation of AMG-carrying virus populations

Taxonomic annotation of the 51,666 AMG-carrying virus populations was established using gene-sharing networks. For each virus population, ORFs were predicted and translated using Prodigal (v 2.6.1) in the metagenomic mode [43]. These protein sequences were then used as input for vConTACT3 (beta mode) (https://bitbucket.org/MAVERICLab/vcontact3/src/master/) to cluster virus populations with NCBI Virus RefSeq (release 220) using default parameters. The previous two versions of vConTACT [44, 45] converted shared protein clusters (PCs) into a distance value based on the hypergeometric formula, and this distance (along with all other pairwise comparisons) was then constructed into a network that was hierarchically clustered and subsequently cut based on a distance threshold derived from *genus*-rank groupings of the ICTV database. In vConTACT3, distances are based on a Jaccard similarity-like distance metric, and instead of a single network, multiple networks at several clustering identities are employed. Optimal distance thresholds (as v2) are identified for each network and for each taxonomic rank from order to genus. By employing these disparate networks and adding in group-specific markers, vConTACT3 is capable of classifying realm, order, family, subfamily, and genus ranks, with varying levels of confidence for each realm.

Virus clusters were considered well supported if they had > 3 virus sequences and had quality and topology scores > 0.3. Only fully resolved clusters were reported. Although giant viruses were not captured from the taxonomic annotation, directly searching for AMG-carrying virus populations that encoded *polB* were identified and considered as giant viruses [43].

### Complete genomes to create mock communities for benchmarking AMG inferences

Because metagenomic assemblies rarely capture complete genomes, we sought to understand how fragmented genomes in our global ocean datasets might impact our understanding of the fraction of virus populations that carried AMGs. To assess this, we downloaded the complete genomes for 295 viruses that were reflective of virus families identified in the GOV 2.0 dataset [15], which included *Caudoviricetes*, *Phycodnaviridae*, *Corticoviridae*, *Inoviridae*, *Iridoviridae*, *Mimiviridae*, *Tectiliviricetes*, and *Herpesvirales.* These 295 reference genomes were then clustered into 89 populations of which 35 virus populations were singletons using established cut-offs (95% ANI over 80% coverage of the genome [14, 38, 39]) with cluster genomes (https://github.com/simroux/ClusterGenomes) and MMSEQS2 (easy cluster) [40].

Genes were then predicted as described above and considered AMGs if they were assigned metabolic modules using DRAM-v (v 1.0.6) [31] with the bit score ≥ 60, as above and used to assess AMG observation power from fragmented genomic sequence data. To appropriately fragment these genomes, we set the length of AMG-carrying virus population fragments falling between the 10th and 90th percentiles of the length distribution of AMG-carrying virus populations observed in our global ocean dataset (see scripts in Data availability). Only virus populations (*n* = 81) with fragments falling within the length cut-off were kept for further analysis. This process resulted in 473 fragments, and AMGs were then assessed for genomic context using the strategy described above. These fragmented genomic data were then pooled to create a mock community in which 231 of the fragments carried AMGs. To mimic the length distribution of AMG-carrying virus populations from the global oceans, the 473 fragments were randomly sampled with replacement resulting in 4000 fragments (see scripts in Data availability).

### Population-based metabolic gene abundance calculations and establishment of pathway context

To calculate the abundance of virus populations, raw sequencing reads of viromes, and prokaryotic metagenomes were cleaned and trimmed using quality control measures as previously described [35]. Cleaned reads from each of the metagenomic samples were non-deterministically recruited to virus populations (*n* = 579,904) using CoverM (v 0.2.0) (https://github.com/wwood/CoverM). Reads were retained if they had ≥ 95% identity and ≥ 75% read coverage, and the trimmed mean was used to remove the top 5% and bottom 5% depths. Contigs with < 70% coverage within a sample were not considered as established in [15]. The coverage was normalized by the total number of reads per metagenome to compare across samples and summed up per paired samples.

While the above population-based abundance estimates use standard approaches, we were concerned about using simple gene-based read-mapping to estimate AMG abundances since the virus and host versions could be similar. Instead, we used normalized coverage of AMG-carrying populations to estimate virus versus host AMG homolog abundances for the 22,779 AMG clusters in the conservative catalog. In doing so, we avoided skewing abundance estimates due to gene-based recruitment issues, while also leveraging the broader genomic context of contigs to best assign the AMG homolog to a virus or cellular origin.

As a first proxy for assessing the extent of potential metabolic reprogramming targeted by AMGs, we placed AMGs encoded by viruses and ocean microbes into metabolic pathway context assigned as “viral” or “microbial.” Contigs assembled from the 127 prokaryote-enriched metagenomes were considered conservatively microbial contigs if they were not identified as virus contigs and were at least 5 kb in length. These putatively assigned “microbial contigs” were further checked using EukRep (v 0.6.7) [46], which identified that 88% of the contigs were of prokaryotic origin, while 12% were of eukaryotic origin. Annotations of the microbial contigs from the KEGG database were retrieved from a previous study [35]. AMGs described above, and their microbial homologs were then mapped to microbial metabolic pathways using Anvi’o (v 7.1) [47] and visualized with iPath3.0 [48]. Pathways were considered complete using the default stepwise-completeness threshold ≥ 0.75 ([47, 49]). For each KEGG Ortholog (KO), we calculated the abundance of genes attributable to viruses or microbes as follows: For viruses, we calculated the abundance by averaging the abundance of AMG-carrying virus populations, whereas for microbial homologs, we used the KO abundance profile of the microbial fraction as established in [35].

Finally, we predicted structures of novel AMGs in the complete metabolic pathways as another window into function. The complete metabolic pathways are indicators of likely “hot-spots” for virus-directed metabolic reprogramming. Among the complete pathways, we identified novel AMGs that encoded dTMP kinase (Tmk, K00943), dihydrofolate reductase (DHFR-TS, K13998), spermidine synthase (SpeE, K00797), and carbamoyl-phosphate synthase large subunit (CarB*,* K01955). Amino acid sequences of the novel AMGs were clustered using MMSEQS2 [35] with the parameters described previously in the AMG identification and novelty section. The representatives were structurally predicted using the Google collaborative AlphaFold notebook (https://colab.research.google.com/github/sokrypton/ColabFold/blob/main/AlphaFold2.ipynb). The predicted 3D structures were visualized with PyMOL (http://www.pymol.org/pymol).

## Results and discussion

### Towards a systematic, global-oceans AMG catalog

**Fig. 1:**
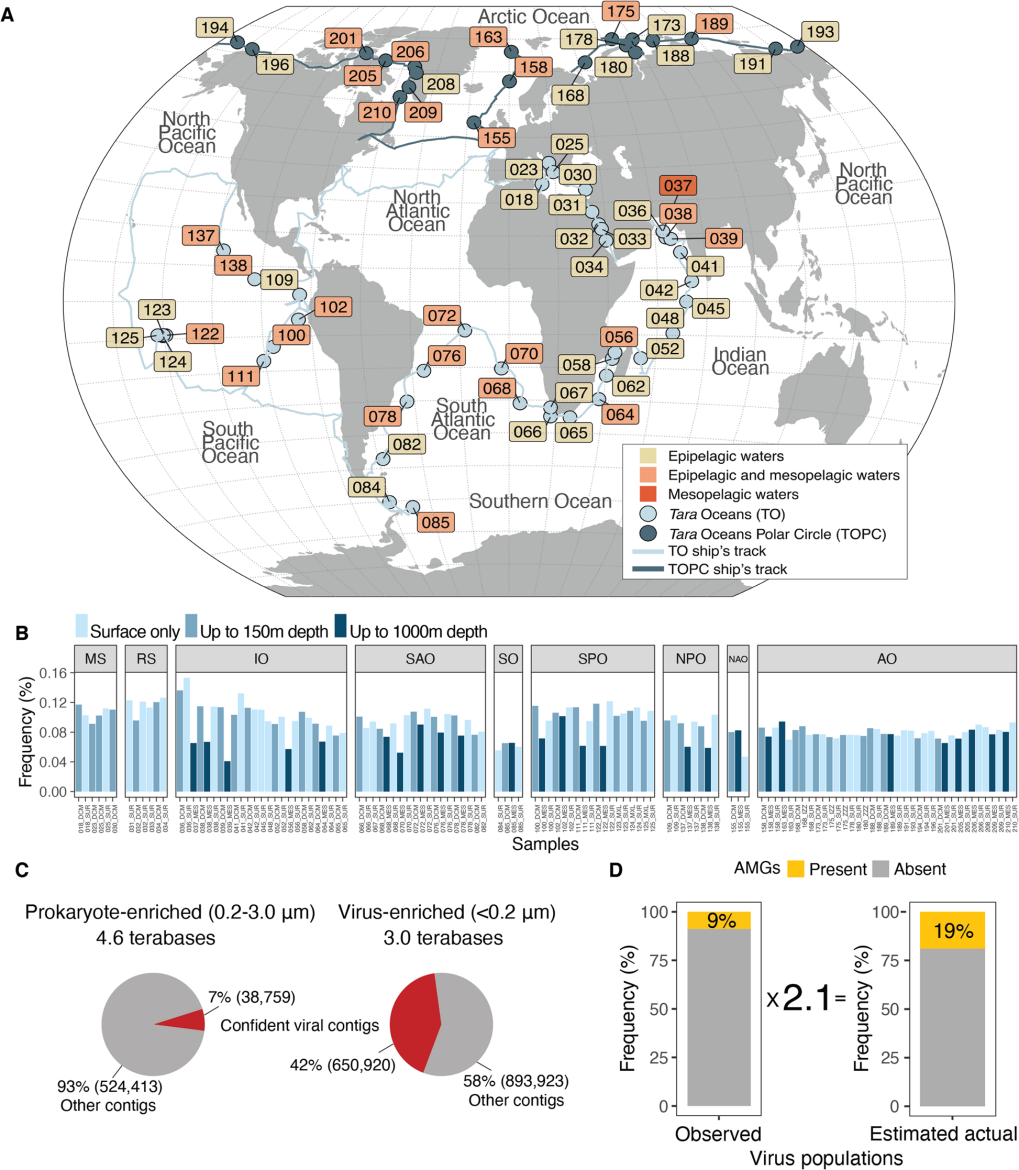
The global ocean virus and auxiliary metabolic genes (AMGs) datasets. **A** Global map showing the location of 62 *Tara* Oceans sampling stations where paired prokaryote- and virus-enriched size-fractionated metagenomes were available. Numbers indicate sampling stations. **B** Bar charts showing the percentage frequency of virus populations (≥ 5 kb) carrying AMGs across the South Pacific Ocean (SPO), North Pacific Ocean (NPO), South Atlantic Ocean (SAO), Indian Ocean (IO), Red Sea (RS), Mediterranean Sea (MS), Southern Ocean (SO), North Atlantic Ocean (NAO), and Arctic Ocean (AO) regions, and across the surface, epipelagic (EPI) and mesopelagic (MES) layers with depths up to 150 m and 1000 m, respectively. **C** Pie charts showing the percentage of confidently identified viruses against the total number of contigs (≥ 5 kb) in prokaryote- and virus-enriched fractions. **D** Stacked bar charts showing the observed proportion of virus populations (≥ 5 kb) carrying AMGs (left) and the estimated actual proportion of virus populations carrying AMGs (right). A conversion factor of 2.1 times was estimated from in silico mock community modeling experiments that sought to extrapolate from observed AMG frequencies in fragmented genomes to the likely actual frequencies in complete genomes (see Methods).We began by developing a robust, semi-automated method to confidently identify virus contigs and virus regions within contigs (e.g., prophages in microbial genomes). While previous studies have developed benchmarked approaches for identifying viruses in ocean metagenome data [13–15], additional rigor is required to ensure that cellular metabolic genes are not incorrectly labeled as AMGs [29]. We used VirSorter2 [37] to identify viruses, which leverages machine learning to identify known and unknown viruses in large-scale metagenomic datasets and has been extensively benchmarked as a top performing virus identification tool [37, 50]. Among 2,108,015 contigs (≥ 5 kb in length) from the prokaryote- and virus-enriched datasets, approximately one-third (*n* = 689,679) were identified as high-confidence virus contigs (see Methods and Fig. S1). These virus contigs were dereplicated by clustering into 579,904 virus populations (approximately equivalent to species-ranked taxa [15, 38, 39]) with a median genome length of 12,608 bp. This increases the number of virus populations by 16% (from 488,130 to 579,904) over those previously described [15]. While 52% (*n* = 303,570) of our dataset was reported in the GOV 2.0 dataset (which relied on VirSorter1) [15], 241,999 virus populations were newly identified by using VirSorter2, and another 37,335 new virus populations were identified by assessing the prokaryote-enriched fraction metagenomes. Comparison of virus populations revealed, only 1% (*n* = 4604) of the virus populations were shared between the prokaryote- and virus-enriched fractions. This supports existing literature, where “virus fraction” populations are different compared to those of the “cellular fractions” [51, 52]

To systematically survey virus AMGs, we used paired prokaryote- and virus-enriched metagenomes (*n* = 254) from 62 stations across the South Pacific Ocean, North Pacific Ocean, South Atlantic Ocean, Indian Ocean, Red Sea, Mediterranean Sea, Southern Ocean, North Atlantic Ocean, and Arctic Ocean, covering polar and non-polar waters, and epipelagic (EPI) and mesopelagic (MES) layers with depths up to 150 m and 1000 m, respectively [14, 15, 34, 35] (Table S3, Fig. 1A–B). While double-stranded DNA (dsDNA) viruses [14, 15] and 243 AMG protein clusters were previously identified and explored in these data [14], the larger virus AMG pool has not been explored owing to challenges in data processing as well as inconsistent AMG identification and annotation between studies. We therefore developed a robust, semi-automated method to confidently identify AMGs from virus contigs and virus regions within contigs (e.g., prophages in microbial genomes).

To interrogate this expanded virus dataset for the presence of AMGs, we adapted a recently proposed AMG identification approach [29] to optimize scalability, maximize functional annotation, and minimize mis-assignment to cellular contigs by combining automated DRAM-v processing [31] with *post-DRAM-v curation rules* (see Methods, Fig. S1). This process identified a permissive, upper-bound AMG-containing catalog (“permissive AMG catalog” Table S4) from 18% (102,369/579,904) of virus populations (Fig. 1C). To obtain a more conservative, lower-bound AMG-containing catalog, we further processed DRAM-v results to remove 13,243 contigs that were potentially beyond prophage genomic boundaries and another 47,673 contigs that may be part of host hypervariable regions and/or genomic islands, as they contained genes/regions related to transposons, integrases, lipopolysaccharides, and/or plasmid stability proteins (Fig. S1).

In total, this additional curation led to the identification of a “conservative AMG catalog” of 86,913 sequences whose virus populations contained on average 1.7 AMGs (range: 1–10 AMGs, Table S5) and represented ~ 9% (51,666/579,904) of total identified virus populations (Fig. 1D). Gene sharing networks [44] were then used to taxonomically classify these AMG-carrying virus populations, although with recent updates in the underlying approaches (see Methods). This revealed 44,988 fully resolved gene sharing network “virus clusters” (approximately genus-level taxa) that assorted predominantly into the class *Caudoviricetes* (*n* = 44,180), but also with representation from other viral classes including the *Tokiviricetes* (*n* = 32) and *Faserviricetes/Huolimaviricetes/Malgrandaviricetes* (genome distance indistinguishable as classified by vConTACT3) (*n* = 3). (Table S6), presumably for artifactual rather than biological reasons. This is because marine RNA viruses predominately infecting eukaryotes were recently shown to encode AMGs [53], although they would not be captured in the DNA-based metagenome datasets we employed. Further, even though giant viruses among have also been shown to carry AMGs [54], they are likely lost during the 0.2 um filtration step, as even in the full dataset they represent only 0.1% (*n* = 766/579,904) of the total identified virus populations based on *polB* marker gene (see Table S4).

To assess which AMGs in our dataset were novel, we compared our catalog against data collected and curated from all available AMG publications (*n* = 101, Table S7). Of these, 64 studies made virus contigs or AMG sequences available during publication or by request, whereas 37 studies had described AMGs, but neither the virus contigs or AMG sequences were provided in the publications and so could not be included in further analyses. Where studies (*n* = 58, Table S7) provided virus contigs or virus genomes, but not AMG sequences, we maximized AMG retrieval by permissively identifying AMGs from the virus contigs (see Methods). This resulted in 22,310 reference AMG sequences from the literature, which clustered into 2467 reference AMG clusters. These reference AMG clusters were then compared against the 22,779 AMG clusters that we had obtained from our 86,913 conservatively identified global ocean AMG sequences (see Data availability), revealing that 32% (7248 out of 22,779) of the AMG clusters identified in our catalog were novel.

Beyond sequence-based novelty, our systematic, global ocean AMG catalog integrates extensive metadata and pathway context to the AMG sequences. The bulk of the 15,531 AMG clusters that had been previously identified by sequence only largely lacked global ocean and/or pathway context (see “Pathway-centric analysis of virus reprogramming in the oceans” section)***.***

### Towards characterizing the fraction of marine viruses that carry AMGs

We next sought to assess what fraction of marine virus populations carry AMGs. Despite there being more than 100 publications describing AMGs in the oceans (Table S7), none have addressed this challenging question owing to: (i) regionally constrained datasets; (ii) genomic data not being evaluated in a population context; and/or (iii) fragmented or incomplete genomic information that are inconsistently evaluated for “virus” origin (see examples Table S7). While the regional and population issues are accounted for by the population-based “vOTU” clustering approach that we applied to the global ocean dataset, the presence of incomplete genomes remains problematic. Specifically, if a fragment of a genome in our dataset lacks an AMG, does that mean the complete genome in the natural sample lacks the AMG, or just that it was missing in our dataset?

To address this, we developed a conversion factor inspired by one employed for microscopy-based viral ecology [55]. This study sought to observe how many cells were *visibly* virus-infected in micrographs and then to extrapolate, via a conversion factor (given that not all stages of infection and all slices of cells will show virus particles), to how many cells are *actually* infected.

Following this logic, we established a conversion factor that extrapolates from the fraction of fragmented-genome-observed AMGs per population to the complete-genome signal. We began by obtaining 295 complete genome sequences representing the following 8 virus families—*Caudoviricetes*, *Phycodnaviridae*, *Corticoviridae*, *Inoviridae*, *Iridoviridae*, *Mimiviridae*, *Tectiliviricetes*, and *Herpesvirales* (Table S8). These were selected as they were reported in the GOV 2.0 dataset [15], had complete genomes from isolates available in GenBank, and had enough representation such that they could be clustered into populations as previously defined [14, 38, 39]. In total, these 295 genomes clustered into 89 virus populations, of which 35 virus populations were singletons (Table S8). We used these data to assess how metagenomics would “see” the AMG content per population by mimicking the observed fragmented genome representation of our global ocean data and screening for AMGs. We focused on 81 out of the 89 virus populations for AMG identification (see “Methods) revealing 70 (86%) of the virus populations carried at least one AMG (average of 11.1 and max of 25 AMGs per population). The 81 virus population genomes were fragmented into 473 fragments, and of these fragments, 231 (49%) contained an AMG (average, 1.9 AMG per fragment; range of AMGs per fragment, 1–20) (Table S8). We randomly sampled these fragments, with replacement, to establish a dataset of 4000 fragments that mirrored the length distribution that we observed in our global ocean AMG-carrying virus populations (Fig. S2). AMG analyses revealed that 40% of these fragments observably contained an AMG (average, 2.2 AMG per fragment; range of AMGs per fragment, 1–11), which is a 2.1-fold underestimate from the 100% of the original, non-fragmented genomes we know contained at least one AMG.

Applying a conversion factor of 2.1-fold to the observed fraction of AMG-containing populations should help to estimate the actual number of AMGs present in the complete genomes. Accordingly, given that we observed at least one AMGs to be present in 9% of virus populations in the conservative AMG catalog, this would represent an *actual* AMG-carrying virus population frequency of 19% (see Methods and Fig. 1C). If the permissive AMG fraction observed of 18% is closer to reality, then as much as 38% of marine virus populations likely contain identifiable AMGs.

### Pathway-centric analysis of virus reprogramming in the oceans

**Fig. 2:**
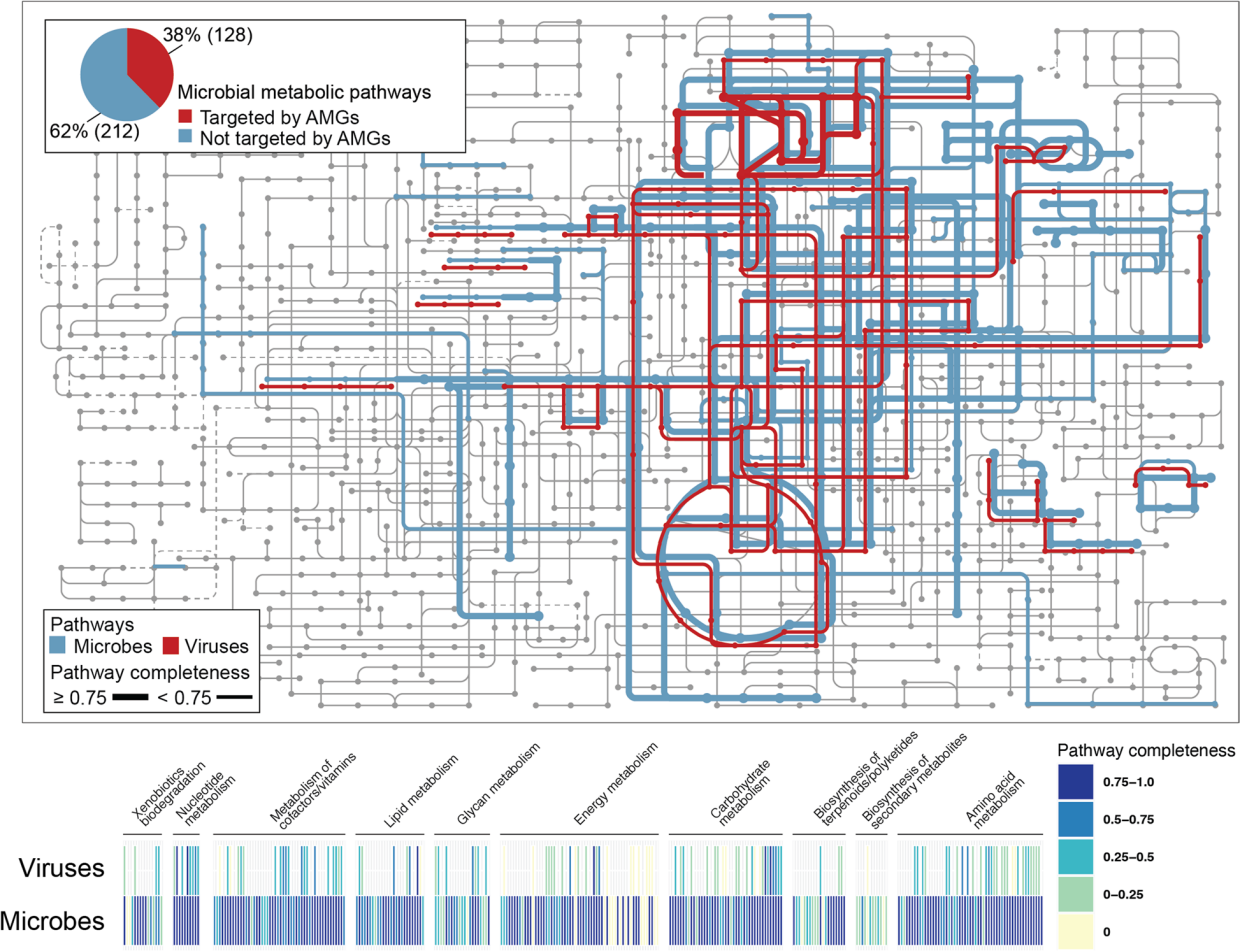
Metabolic pathways detected in global ocean microbes and viruses

We next asked which metabolic pathways were present in microbes throughout the global oceans. Using a stepwise pathway-centric approach, where a step represents either one metabolic reaction or a branch point in the pathway [47] (see Methods), we determined the identifiable pathways (and their completeness) encoded in microbes from the prokaryote-enriched fraction (see Methods). Overall, this revealed 340 metabolic pathways, 205 of which were considered complete or near-complete (completeness ≥ 0.75) [56] (Fig. 2 and Table S9). At a high level, these pathways are involved in carbon, energy, nucleotide, lipid, and amino acid metabolism (Fig. 2 and Table S9). Interestingly, these included three metabolic pathways not usually associated with ocean-dwelling microbes [53, 57–59] (Fig. S3).

KEGG metabolic pathway map (grey) annotated with global ocean pathways detected on microbial (blue) or virus (red) contigs. The virus contig data shown here were derived from the conservative AMG catalog (see Fig. S3 for the permissive AMG catalog findings). Nodes represent chemical compounds, whereas edges (lines) represent a series of enzymatic reactions with line thickness representing pathway completeness. Inset pie chart summarizes the fraction of marine microbial pathways known that either had virus-encoded AMGs detected (red) or not (blue). Bar chart below the metabolic pathway map which summarizes the metabolic pathway completeness in the virus and microbial contigs respectively.

We next wondered what fraction of these microbial metabolic pathways are targeted by virus AMGs. Using the conservative AMG catalog, we found that 128 of the 340 microbial metabolic pathways were targeted (Fig. 2 and Table S10). This included 119 pathways where < 75% of the steps had cognate AMGs (hereafter an “incomplete” pathway) and 9 pathways in which ≥ 75% of the steps were recovered in our AMG catalog (note: AMG-complete pathway signals are not derived from the same virus, but from aggregated community-wide signals; Table S6). The 119 incomplete pathways were associated with carbohydrate, energy, lipid, amino acid, glycan, cofactors, vitamins, terpenoids and other secondary metabolites, and degradation of polyketides (Table S10). When using the permissive AMG catalog, viruses targeted 199 pathways including 45 complete pathways (Fig. S4 and Table S11). Interestingly, within the permissive catalog, we identified AMGs associated with rare metabolic functions that included, xenobiotics biodegradation, drug resistance, and pathogenicity signatures (Table S11). In both the conservative and permissive AMG scenarios, these findings suggest virus-encoded AMGs could result in broad virus reprogramming of microbial metabolism if expressed during infection—particularly considering approximately one in three cells are infected at any given time [4].

**Fig. 3:**
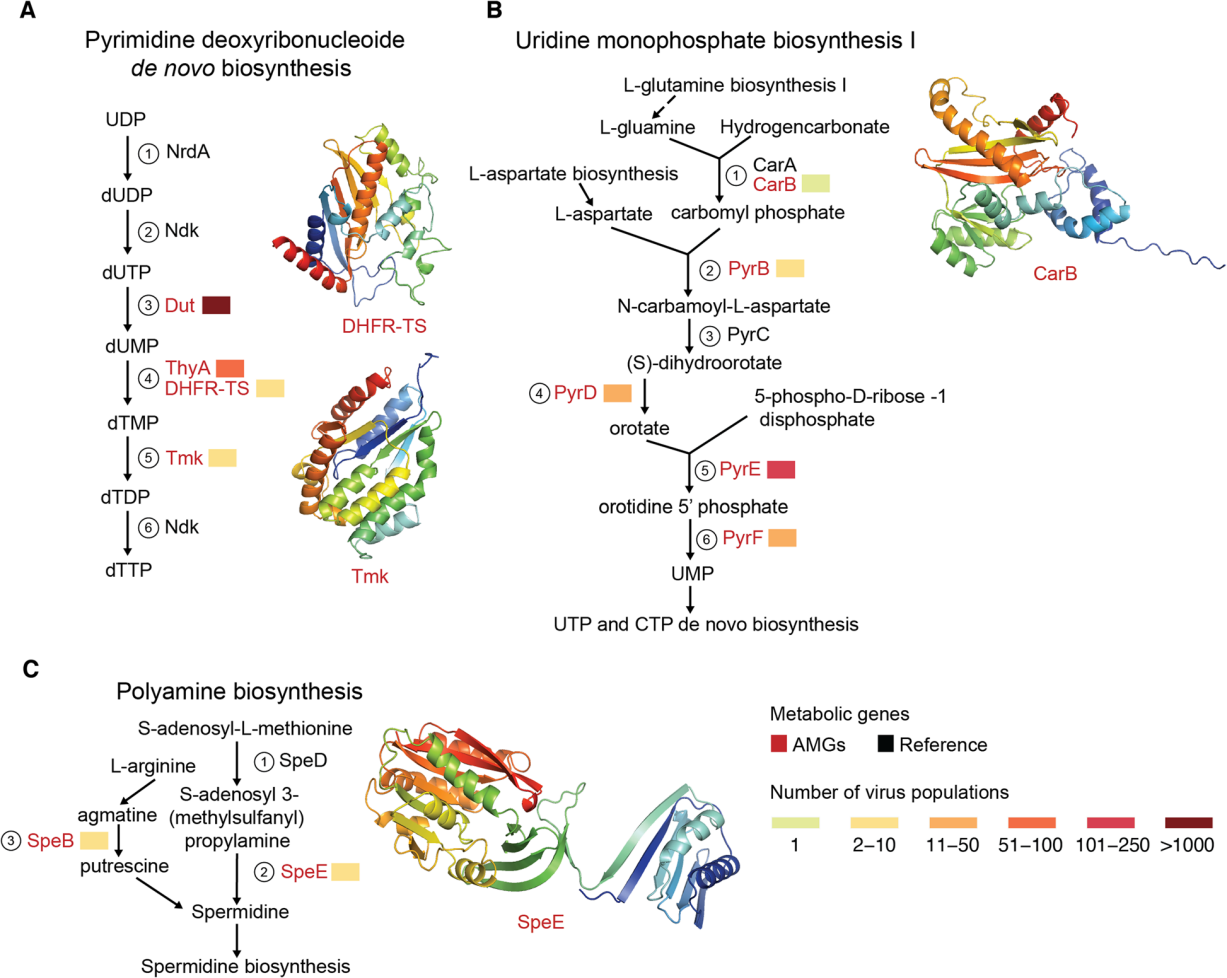
Complete metabolic pathways targeted by novel AMGs

We were particularly intrigued by the nine complete pathways in our conservative AMG catalog, whose completeness implies that they are “hot-spots” for virus-directed metabolic reprogramming. These nine pathways were associated with carbohydrate metabolism (three pathways—pentose phosphate pathway, reductive pentose phosphate and PRPP biosynthesis pathway), amino acid metabolism (two pathways—methionine degradation and polyamine biosynthesis pathway), lipid metabolism (one pathway—fatty acid biosynthesis pathway), and nucleotide metabolism (three pathways—pyrimidine deoxyribonucleotide biosynthesis, inosine monophosphate biosynthesis, and uridine monophosphate biosynthesis pathway) (Fig. S5). Though many of these AMGs have been previously reported (see Tables S1 and S2), they were only rarely described in the context of a pathway [10, 18–20] and never in the context of the virus population encoding them. We found that some pathways were encoded by a single virus population (e.g., *prsA* within the phosphoribosyl diphosphate biosynthesis pathway), whereas others were encoded by > 1000 virus populations (e.g., dut in the pyrimidines) (Fig. 3 and Fig. S5).

The varying number of populations encoding AMGs targeting various reaction steps in a pathway likely indicate niche differentiation among virus populations that are under different infection lifestyles, hosts, or environmental ecosystems. Moreover, we hypothesize that there is a relationship between the breadth of microbial taxa that encode those metabolisms and the number of virus populations that encode AMGs within those pathways, given marked differences in metabolic pathways among microbial phyla identified in gut microbes [60], as well across in carbon fixation pathways in bacteria and archaea [61].

Beyond evaluating the number of virus populations that encoded AMGs, we were also interested in whether these complete pathways encoded novel AMGs. Of the nine complete pathways, we found that three encoded at least one novel AMG in one of the reaction steps. These were associated with nucleotide (two pathways—pyrimidine deoxyribonucleotide biosynthesis and uridine monophosphate biosynthesis) and amino acid metabolism (one pathway—polyamine metabolism) (Fig. 3).

Of the 128 ocean virus AMG-targeted pathways, nine were targeted completely such that an AMG was observed for every reaction step in the known metabolic pathway. The six metabolic pathways completely targeted by previously known AMGs, though only three (pentose phosphate pathway, phosphoribosyl diphosphate (PRPP) biosynthesis pathway and fatty acid metabolism pathway) have been formally documented as AMG-targeted [9]. We identified three other metabolic pathways that are completely targeted by AMGs where we designate an AMG (red) from the current study and/or published studies to document AMG targeting in these metabolic pathways. These three pathways include the following: (A) pyrimidine deoxyribonucleotide de novo biosynthesis I; (B) uridine monophosphate biosynthesis I; and (C) polyamine biosynthesis. For each novel AMG from this study, we obtained AlphaFold 3D structures, with Tmk, DHFR-TS, and SpeE having very high model confidence (pLDDT ≥ 90) and CarB having low model confidence (50 ≤ pLDDT < 70). References (black) are enzymes involved in reaction steps per pathway as defined in the MetaCyc database. Reaction steps are indicated via circled numbers; those lacking a number are independent of enzymatic reactions. The other six metabolic pathways completely targeted by previously known AMGs are shown in Fig. S5.

The first and second pathways involved three novel AMGs associated with nucleotide metabolism as related to the pyrimidine deoxyribonucleotide biosynthesis and uridine monophosphate synthesis pathways (Fig. 3A–B). These new AMGs encode dihydrofolate reductase (DHFR-TS, K13998) and dTMP kinase (*tmk*, K00943) in the pyrimidine deoxyribonucleotide biosynthesis (Fig. 3A), and carbamoyl-phosphate synthase large subunits (*carB,* K01955) in uridine monophosphate synthesis (Fig. 3B). As they are new AMGs, we also evaluated 3D structural predictions, which for the most part confidently supported these functional assignments (confidence level pLDDT ≥ 90, except for *carB* that had a confidence level 50 ≤ pLDDT < 70; Fig. 3A–B). If functional, then during DNA synthesis, the dihydrofolate reductase would convert dUMP to dTMP [62] and the dTMP kinase would convert dTMP to dTDP [63], whereas CarB would synthesize arginine and pyrimidine biosynthetic precursor molecules [64].

Viruses are well-known to reprogram nucleotide metabolism (Table S1 and S2), as nucleotides are essential for virus genome replication, the second most energetically expensive process during infection after translation [65]. Presumably, phages carry nucleotide synthesis AMGs to ensure the production of energy-demanding nucleotides regardless of the status of the host cell or its surrounding environment. Alternatively, some bacteria deplete their own nucleotide pools as a defense mechanism during phage infection [66]. In this scenario, viruses may carry these AMGs to overcome host defenses.

The third complete pathway was the one-step polyamine biosynthesis pathway, where we identified the AMG spermidine synthase (*speE*, K00797). Though there have been reports of *speE* in giant viruses [38], this is the first time in phages, and the enzyme’s functional prediction is well-supported by a confident structural prediction (pLDDT ≥ 90) (Fig. 3C). Spermidine has previously been shown to enable both genome replication and packaging in eukaryotic viruses [67–69]. Polyamines increase messenger RNA production and translation to trigger de novo production and utilization of nitrogen. This modulation of the host’s assimilated nitrogen pools favors virus genome replication and packaging into the capsid [54]. During virus infection in eukaryotes, host cells reduce spermidine production to inhibit virus replication [54]. Similarly, phages encoded polyamine-synthesis AMGs may result from a disproportionately higher need for nitrogen-rich resources, including amino acids, than can otherwise be obtained from recycling host proteins [70], while also serving as a mechanism to evade host suppression during infection.

### Virocell metabolic pathways augmented by AMGs

**Fig. 4:**
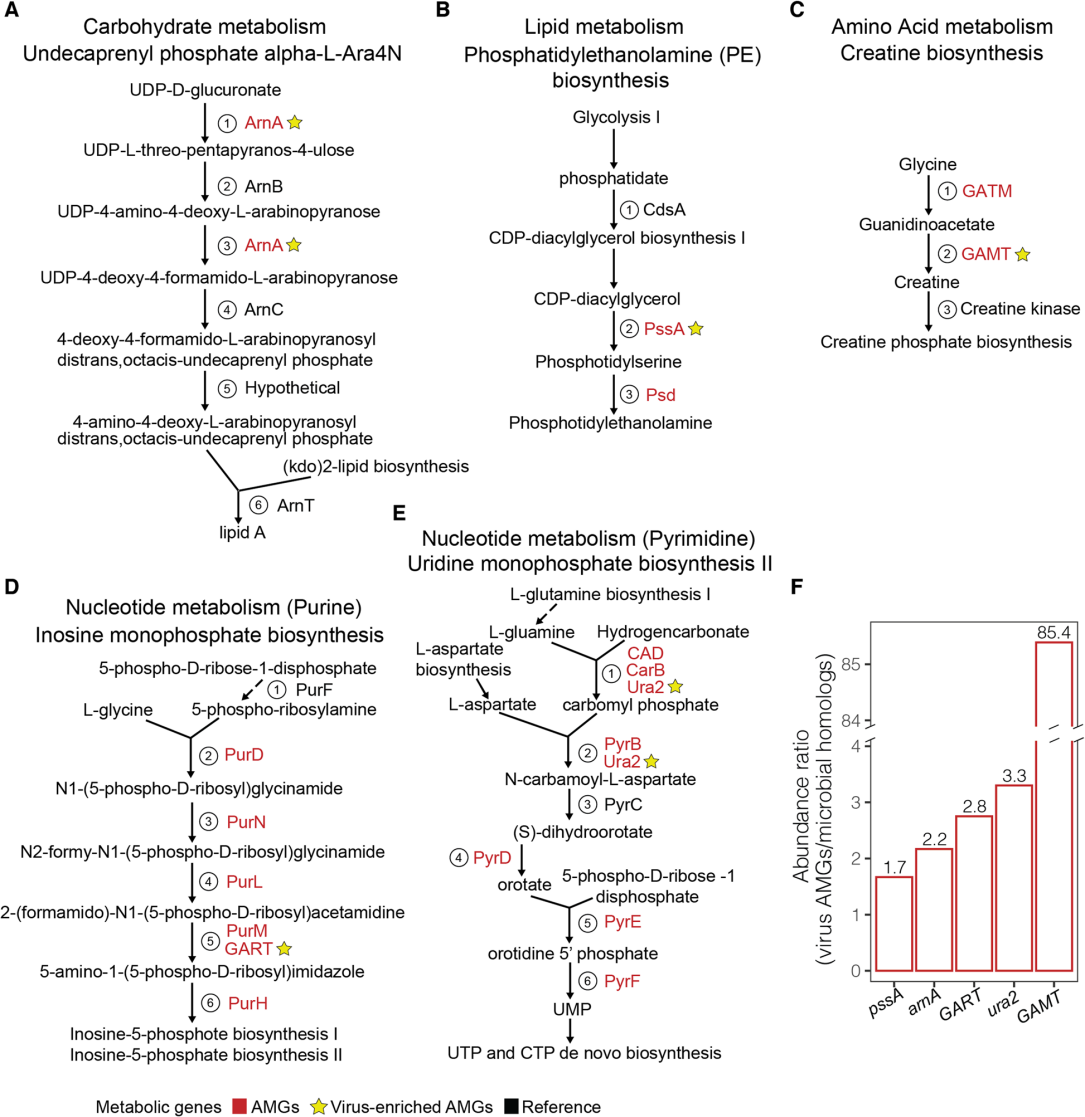
Metabolic pathway steps augmented by AMGs. Through enrichment analysis, the abundance of AMGs and their microbial homologs were assessed in virus versus microbial contigs across 146 AMGs with KEGG annotation. Shown here (**A–E**) are microbial metabolic pathways where at least one step was enriched for viruses, as determined by the abundance of a virus-encoded AMG (red text) being higher than that of their microbial homologs. Virus-enriched AMGs are starred (yellow). References (black) are enzymes involved in reaction steps per pathway as defined in the MetaCyc database. Reaction steps are indicated via circled numbers, and those lacking a number are independent of enzymatic reactions. **F** Summary data for virus-enriched AMGs and their levels of enrichment (the number on top of each bar) are provided as a bar chart

To test the hypothesis that AMGs target key steps in metabolic pathways [10, 65], we sought to calculate the abundance of genes encoding each reaction step to provide a quantitative view of on which reaction steps were targeted. To this end, we calculated the ratio between the abundance of virus-encoded AMGs (*n* = 146) against that of microbial homologs (see Methods). We inferred the former by read-mapping against the representative sequence from each AMG-carrying virus population, rather than directly read-mapping to the AMG (Table S12, see Methods). We chose this approach to avoid ambiguous source inferences since short-reads can rarely differentiate viruses from microbial homologs [32, 71] Microbial homolog abundances were derived as previously reported [35] (see Methods; Table S13). Only five virus AMGs were found to be more abundant than their microbial homologs (Fig. 4A–E).

In carbohydrate metabolism, we identified one AMG, UDP-4-amino-4-deoxy-L-arabinose formyl transferase (*arnA*, K10011), that was virus-enriched (Fig. 4A) and is one among the four genes involved in making lipid A phosphates [67] (Fig. 4A). These AMGs and have not been previously reported in marine viruses, with *arnA* being 2.2-fold more abundant in viruses than in the microbial homologs (Fig. 4F). Biogeographically, this AMG was distributed in the Arctic Ocean and South Atlantic Oceans and present in the surface and mesopelagic water columns (Table S14). In bacteria, lipid A is a component of lipopolysaccharides (LPS) and forms part of the active components involved in host immune responses [72]. Bacteria have evolved mechanisms to alter their LPS membrane structure in response to environmental cues [73, 74], resulting in resistance against antimicrobial compounds and antibiotics [75]. Furthermore, temperature has been found to induce changes in bacteria membrane architecture [76, 77]. It is thus possible that the cold waters of the Arctic and South Atlantic Oceans act as environmental triggers to elevate ArnA abundance, leading to remodeling of the bacterial membrane to achieve optimal survival conditions for the virus and host. In lipid metabolism, one of the four genes involved in the phosphatidylethanolamine (PE) biosynthesis pathway CDP-diacylglycerol–-serine O-phosphatidyl transferase (pssA, K00998) was enriched 1.7-fold over the microbial homolog (Fig. 4B, Fig. 4F, and Table S13). *PssA* was not previously identified in marine phages, and here, biogeographically, *pssA* was enriched in the Indian Ocean and confined to deep chlorophyll maximum (DCM); *psd* abundances were, however, negligible across the global oceans (Table S14). *PssA* and *psd* presumably catalyze the de novo synthesis of phosphatidylserine, which is a membrane phospholipid present in both eukaryotes and prokaryotes [78, 79]. In eukaryotic and some prokaryotic cells, phosphatidylserine residues are a global immunosuppressive signal that triggers cell death (i.e., apoptosis) [78, 80]. However, under normal physiological conditions, phosphatidylserine facilitates tolerance and prevents local and systemic immune activation [80]. Therefore, viruses carrying AMGs associated with phosphatidylethanolamine biosynthesis pathway (*pssA* and *psd*) could hijack the host immune response via phosphatidylserine to enable their infection and replication. Although it is unclear why *pssA* was distributed only in the Indian ocean, we speculate that the oligotrophic nature of this ocean region, characterized by elevated levels of lytic viruses [81], would likely result in evolution of microbial host defense mechanism, such as the phosphatidylethanolamine (PE) biosynthesis pathway to evade virus infection. Virus AMGs likely counter these defense mechanisms, enabling successful infection and replication within the microbial hosts.

In amino acid metabolism, one of the three genes involved in creatine biosynthesis was enriched 85.4-fold over its microbial homologs; this AMG was guanidinoacetate N-methyltransferase (GAMT, K00542). This was the greatest enrichment we observed, indicating that this AMG is likely a critical metabolic step for infecting viruses (Fig. 4F).

Biogeographically, all nine virus populations carrying GAMT were confined to the Arctic Ocean, while six of the nine virus populations carrying GAMT were present in the DCM and mesopelagic (MES) water columns, with the remaining three present in the surface water column (Table S14). During creatine biosynthesis, GATM is involved in the transfer of amidino groups to glycine resulting in the formation of guanidinoacetate, while GAMT catalyzes the transfer of methyl groups from *S*-adenosyl-L-methionine to guanidinoacetate leading to the formation of the creatine [82]. In vertebrates and sponges, creatine acts as a buffer in tissues where ATP demand exceeds synthesis, allowing ATP to be shuttled to high consumption sites [83, 84]. Among both DNA and RNA viruses, low oxygen conditions (hypoxia) greatly inhibit replication under experimental culture conditions [85]. To increase ATP production in hypoxic conditions, viruses trigger reversible reactions in which creatine kinase splits creatine phosphosphate to creatine and ATP [85, 86]. We hypothesize that GAMT enrichment might enable the continuous production of creatine in low oxygen water columns within the Arctic Ocean regions.

In nucleotide metabolism, two gene of the fifteen AMGs, were > 2.8- and > 3.3-fold virus-enriched in phosphoribosylamine–glycine ligase (GART, K11787) for purine and carbamoyl-phosphate synthase (ura2, K11541) for pyrimidine synthesis, respectively (Fig. 4D–F and Table S13). Biogeographically, *GART* was enriched in the Arctic Ocean and Red Sea, while *ura2* was found in the Arctic Ocean (Table S14). The two AMGs were distributed in the DCM and MES water columns, respectively (Table S14). *GART* is reported for the first time in marine viruses. In bacteria, GART reversibly catalyzes glycine ligation in de novo purine biosynthesis [87]. Ura2 catalyzes the biosynthesis of the precursor used in the production of pyrimidine nucleotides and L-arginine (in vertebrates [88]) through a multistep process [89] and has previously been reported in marine viruses [14]. We hypothesize that the novelty of the *GART* is probably a reflection of the rare microbial taxa that viruses infect in the Arctic and Red Sea regions. Together, these observations add to a growing body of literature showing that phages modulate de novo nucleotide metabolism (Table S1 and S2).

Overall, we found that viruses target key steps within a metabolic pathway, though the mechanism of how and why this occurs is uncertain. We hypothesize that this could be due to the thermodynamic or kinetic benefit of targeting a particular bottleneck step associated with flux in a given pathway. While the mechanisms and “selection criteria” for the enrichment of AMGs in a metabolic pathway remain unclear, these AMGs represent ideal targets for further mechanistic study through community metabolic modeling [90, 91] or modeling time-resolved multi-omic virocell infection experimental data [8, 92].

## Limitations, future opportunities, and conclusions

The advent of genome-resolved metagenomic studies, the availability of global ocean datasets, and increasingly powerful and nuanced bioinformatics workflows have heralded a new era of AMG discovery, with more than 100 papers now describing AMGs across diverse systems (Tables S1 and S2). We sought to develop a scalable and systematic approach to survey AMGs across the global oceans. The resulting curated marine AMG catalog and global analyses represent a critical resource for predictive models to assess how virus-encoded AMGs impact ecological processes and biogeochemical cycles [93]. “Though giant viruses are increasingly known for their [94, 95] this global ocean AMG catalog is primarily derived from prokaryotic viruses since the giant viruses are under-represented in virome fraction metagenomes (the particles are predominantly larger than the 0.2um filtration pore size) and our virus identification tool (VirSorter2), which was developed and benchmarked for prokaryotic-viruses identification with only minimal attention to establishing a giant-virus classifier due to lack of expertise available at the time [37].

Ecological and metabolic analyses of marine AMGs face several challenges. Current datasets are mostly derived from short reads, resulting in fragmented genomes that may confound virus versus cellular sequence assignments, particularly in hypervariable genomic regions where viruses likely sample sequence space to obtain new AMGs. Thus, our conservative AMG dataset likely underestimates AMG frequencies in the ocean. Adopting long-read sequencing technologies will improve the genomic context [96–99] and accuracy of AMG virus-versus-host assignments, allowing the prediction of virus taxonomy and hosts. Yet even with improved virus genome catalogs, in most cases, we are unable to link the many newly discovered viruses to hosts directly. While PCR-based approaches offer targeted solutions [100, 101], community-wide inferences using emerging technologies such as proximity ligation [102–104] and single-cell genomics [105] may provide more accurate and broadly valuable virus-host interaction predictors. Proximity ligation data might also be used to link fragmented short-read-assembled contigs to better capture hypervariable genomic island regions (e.g., cell surface modulation genes and mobile genetic elements) that we conservatively excluded from our current analyses. Functional annotation remains challenging in two ways: (i) per-gene annotations often offer no more than “hypothetical protein” for many open reading frames (though large-scale efforts like [106] may help), and (ii) even where functions are annotated, only 3% of our identified AMGs could be mapped to pathways in the KEGG database, which is heavily based on well-studied model organisms. This leaves many potential AMGs unidentified and/or without obvious pathway context and thus an area of opportunity for future AMG-focused work. Finally, some AMGs are deceptive in their function (e.g., a divergent “*pebA*” was experimentally shown to perform the function of cellular *pebA* and cellular *pebB* [107]) such that functional studies will be needed to clarify the metabolic roles of more divergent putative AMGs. As such obstacles are overcome, our systematic and global oceans AMG catalogs can be revisited to further explore ecological and metabolic patterns of AMGs within a broader mechanistic metabolic modeling framework.

Ultimately, we are deep into the era of the Anthropocene [108], and thus it is critical that we better monitor microbial roles in planetary functioning. To do so, we will require new modeling approaches and comprehensive AMG catalogs to more explicitly bring viruses (via AMG-modulated metabolic pathway manipulations) into biogeochemical models that are increasingly needed to develop policy and predictions about the future of our planet [109].

### Supplementary Information

Supplementary Material 1.

## Data Availability

Assemblies from the virus-enriched fraction used in the identification of AMGs are available in iVIRUS. While assemblies and genes from the prokaryote-enriched fraction can be accessed from here: https://www.ebi.ac.uk/biostudies/studies/S-BSST297. AMG sequences, populations and contigs identified in this study can be accessed here: https://doi.org/10.5281/zenodo.12668289.
