# Supplementary material for: Prokaryotic-virus-encoded auxiliary metabolic genes throughout the global oceans
Source: Microbiome. 2024 Aug 29;12:159. doi: 10.1186/s40168-024-01876-z (PMC11360552; doi:10.1186/s40168-024-01876-z)
Supplement: Supplementary file 2 — Supplementary Material 1. [file 40168_2024_1876_MOESM1_ESM.docx]

**
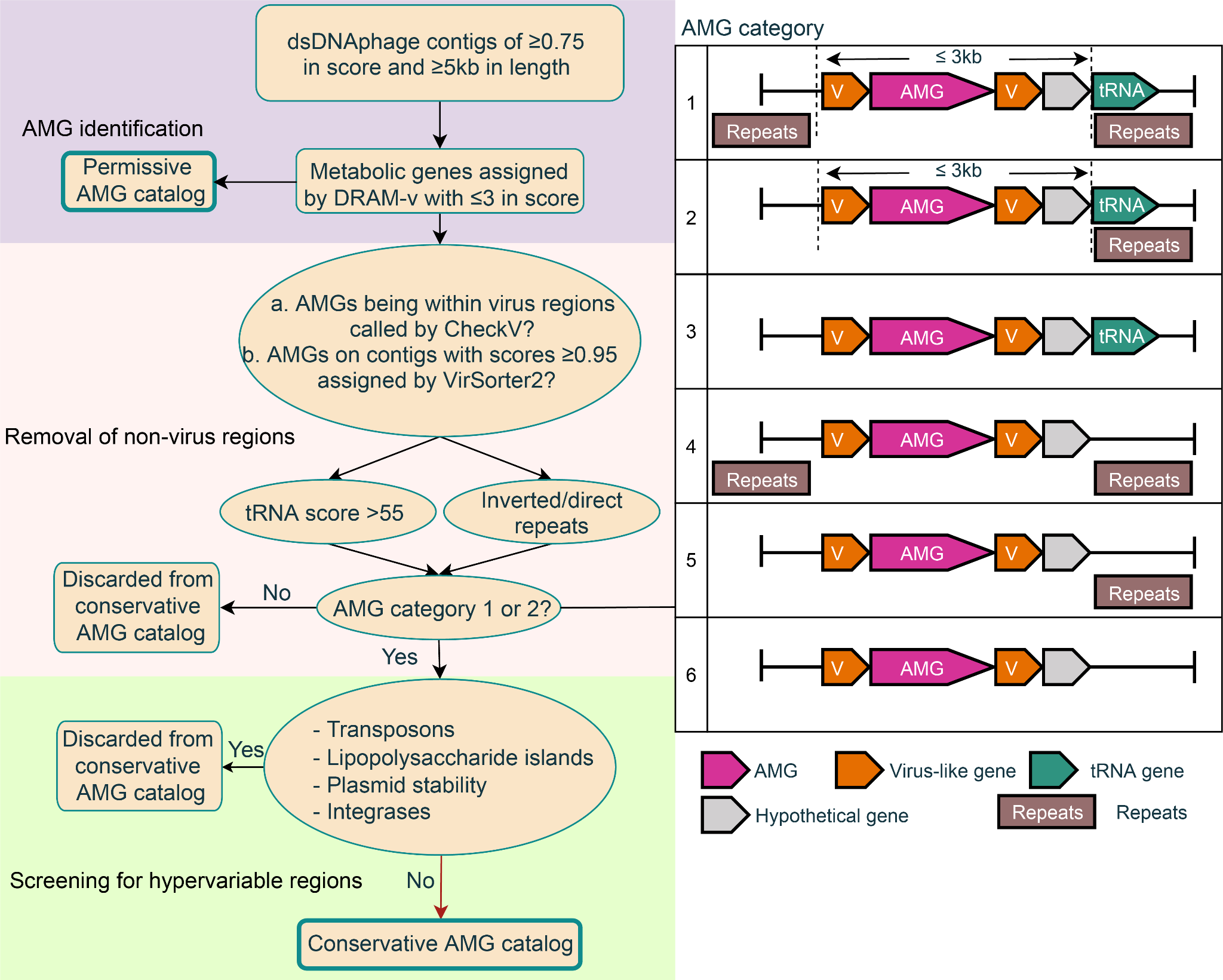
**

**Figure S1. Schematic representation of the AMG identification and curation pipeline.** Left panel: Flow diagram showing steps in identifying and curating AMGs. The workflow used a combination of both automated and manual curation steps as follows. Automated AMG identification was carried out by DRAM-v, resulting in the permissive AMG catalog . Additional automated steps included the removal of non-virus regions by CheckV, detection of tRNA regions by tRNAscan-SE and prediction of inverted and direct repeats by EMBOSS. Manual curation involved the removal of contigs annotated by DRAM-v to carry the following proteins, “*transposons*”, “*lipopolysaccharide* *island*”, “*plasmid stability*”, “*integrase*”, resulting in the conservative catalog (see **Methods**). Right panel: categories designated to AMGs based on gene neighborhoods in the contig. Genes are color coded to represent AMGs (pink), viral-like genes (orange) determined by VirSorter2 [38], tRNA genes (green), hypothetical genes (gray). Inverted and direct repeats are shown in brown rectangles.


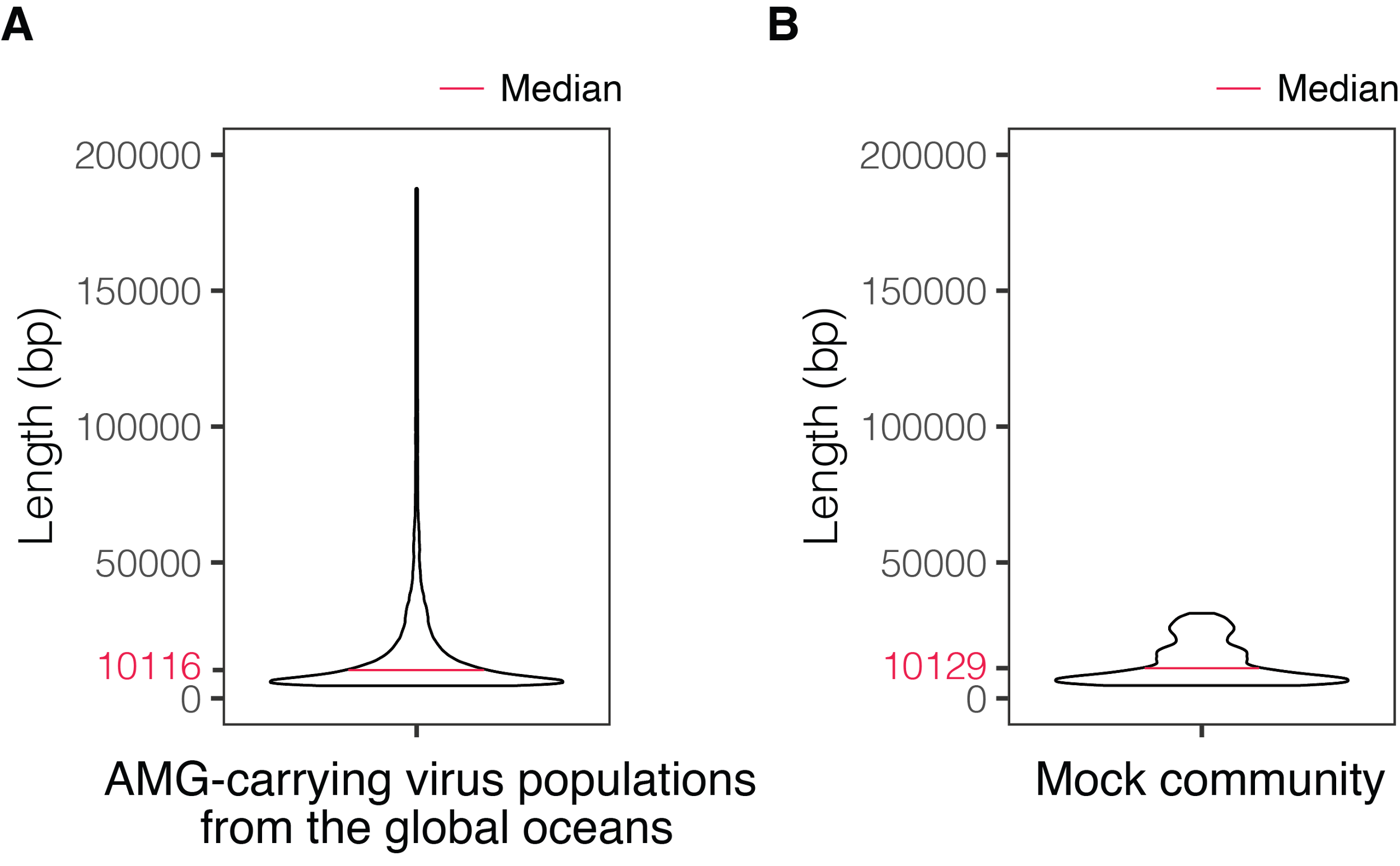


**Figure S2. Mock community experiments to establish an “observed” AMG population conversion factor.** To estimate a conversion factor between the observed fraction of virus population genome fragments that contain AMGs as compared to the actual complete genomes signal, we established an *in silico* mock community experiment from 295 complete genomes across 8 viral families (**see Methods**).

**(A)** Violin plot showing the length distribution of AMG-carrying virus populations from the global oceans.

**(B)** Violin plot showing the length distribution of fragmented genomes in the mock community.

**
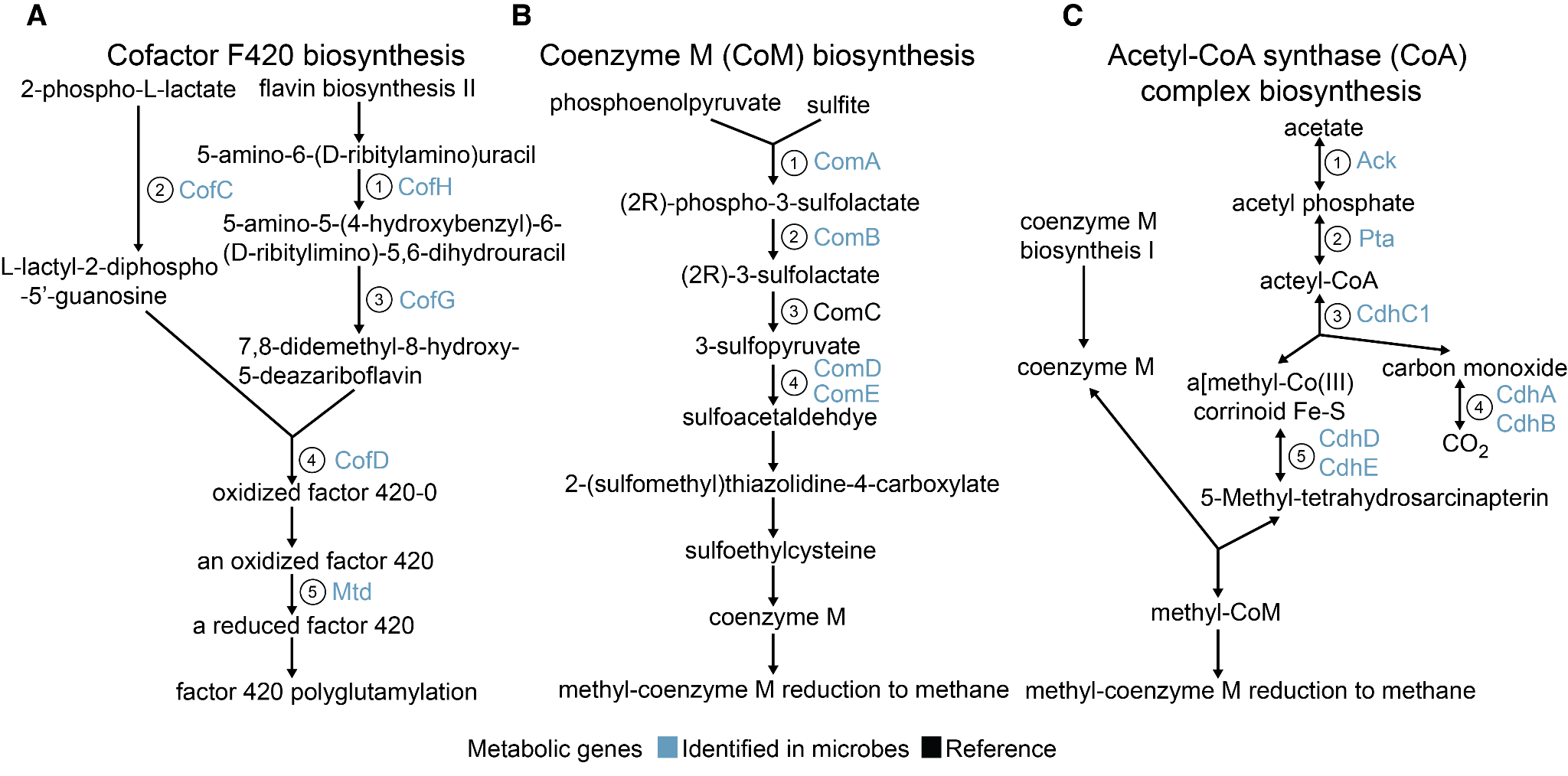
**

**Figure S3. Examples of three of the 340 complete metabolic pathways found in ocean microbes.**

To highlight how a pathway-centric investigation can inform our understanding of ocean metabolism, we initially focused our attention on a particular set of unexpected pathways present in the microbial fraction. The three pathways were annotated as being involved in – **(A)** Cofactor F420 biosynthesis, **(B)** Coenzyme M (CoM) biosynthesis, **(C)** Acetyl-CoA synthase (CoA) complex biosynthesis**.** The first two pathways are more typically associated with archaeal methanogens whereas the latter pathway is known to be widespread in bacteria. Enzymes identified in ocean microbes are delineated with blue text, while references (black) are enzymes involved in reaction steps per pathway as defined in the MetaCyc database. Reaction steps are indicated via circled numbers, and those lacking a number are independent of enzymatic reactions. The complete set of observed microbial metabolic pathways are documented in **Table S9**. The first two pathways are more typically associated with archaeal methanogens [55-57], and not usually associated with ocean-dwelling microbes, while the latter pathway is known to be widespread in bacteria [58].


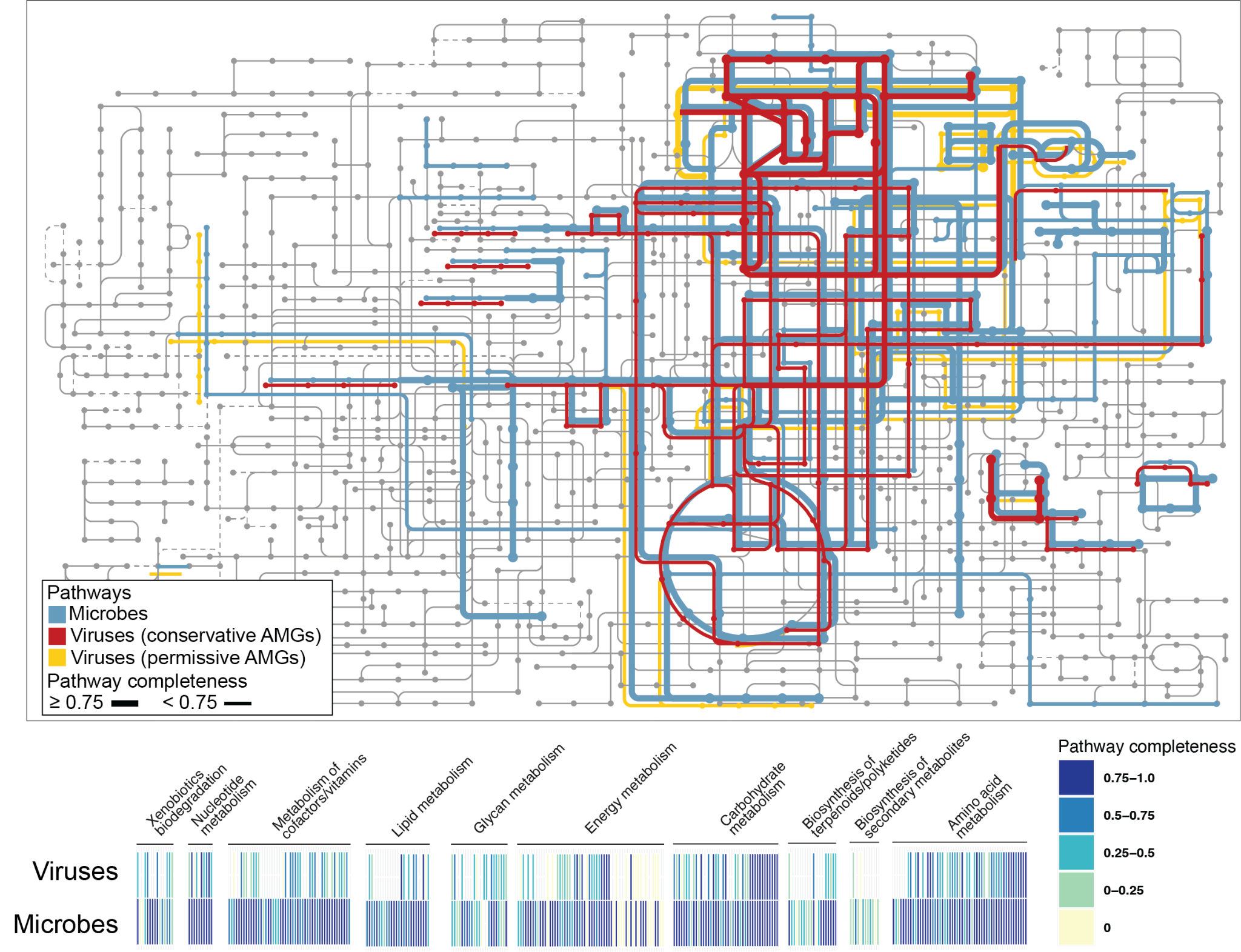


**Figure S4. Metabolic pathways of microbes and viruses from the global oceans.**

KEGG metabolic pathway map (gray) annotated with global ocean pathways detected on microbial (blue) or virus (red and yellow) contigs. The virus contig data shown here were derived from the conservative and permissive AMG catalog. Nodes represent chemical compounds, whereas edges (lines) represent a series of enzymatic reactions with line thickness representing pathway completeness. Barchart below the metabolic pathway map which summarizes the metabolic pathway completeness in the virus and microbial contigs respectively.


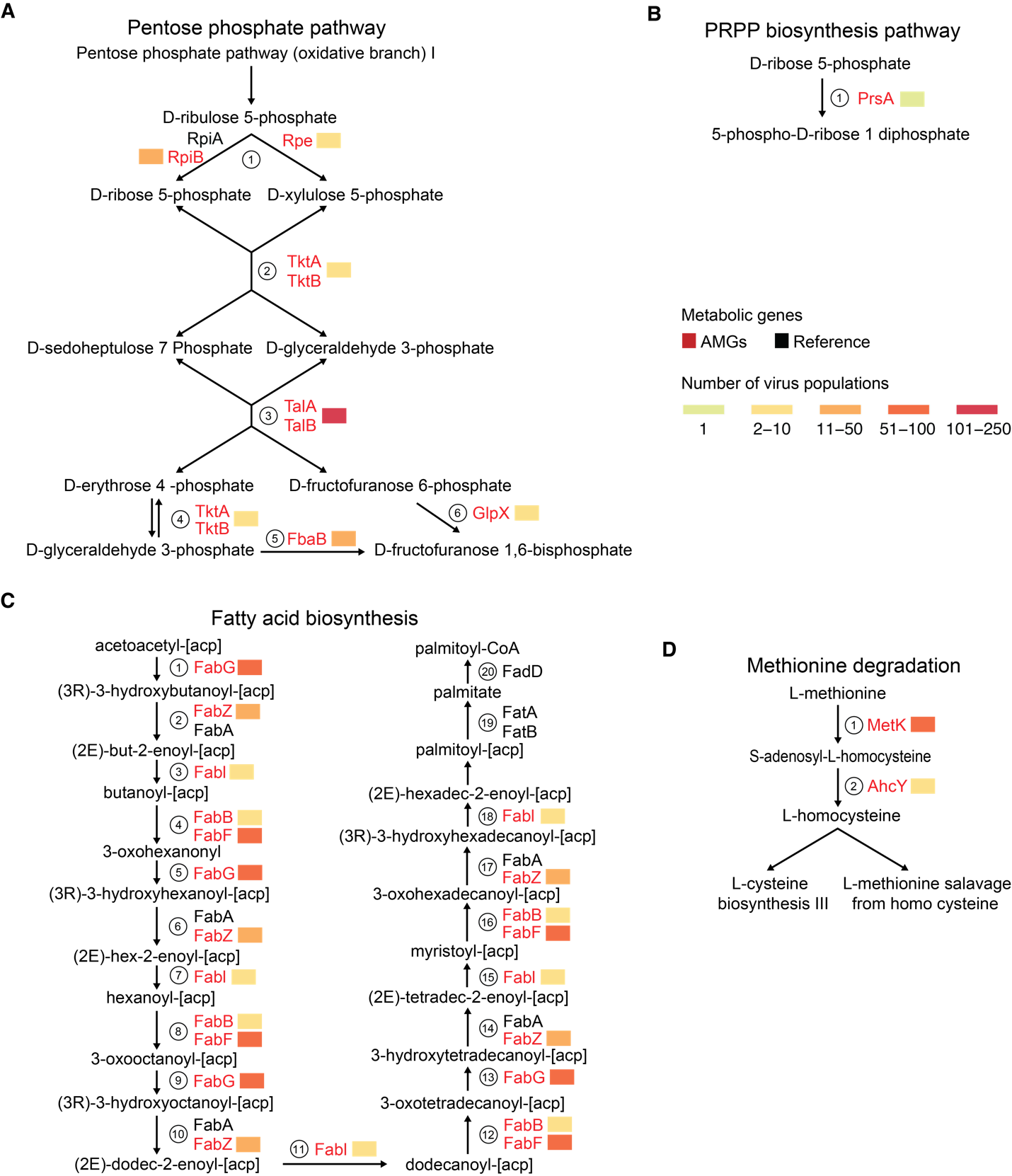


**Figure S5. Complete metabolic pathways targeted by AMGs but have previously been identified.**

**(A)** Pentose phosphate pathway, **(B)** Phosphoribosyl diphosphate (PRPP) biosynthesis pathway, **(C)** fatty acid biosynthesis and **(D)** methionine degradation with the AMGs shown in red, reaction steps indicated in numbers, and number of virus populations marked next to each AMG. References (black) are enzymes involved in reaction steps per pathway as defined in the MetaCyc database. Reaction steps are indicated via circled numbers, and those lacking a number are independent of enzymatic reactions.
